# Associations of depression and depressive symptoms with preeclampsia: results from a Peruvian case-control study

**DOI:** 10.1186/1472-6874-7-15

**Published:** 2007-09-27

**Authors:** Chunfang Qiu, Sixto E Sanchez, Nelly Lam, Pedro Garcia, Michelle A Williams

**Affiliations:** 1 Center for Perinatal Studies, Swedish Medical Center, 1124 Columbia Street, Suite 750, Seattle, WA 98104, USA; 2 Health Direction V Lima City: Jr. Antonio Raymondi 220, La Victoria, Lima, Peru; 3 Materno Perinatal Institute, Jr. Antonio Miroquezada 941, Barrios Altos, Lima, Peru; 4 Department of Epidemiology, University of Washington, School of Public Health and Community Medicine, 1959 NE Pacific Street (HSB F-343; Box 357236), Seattle, WA 98195, USA

## Abstract

**Background:**

Preeclampsia involves endothelial dysfunction, platelet dysfunction/activation and sympathetic over-activity similar to cardiovascular disorders (CVD). Depression, an independent risk factor for progression of CVD, was found to be associated with an increased risk of preeclampsia among Finnish women. We examined the relation between depression/depressive symptoms and preeclampsia risk among Peruvian women.

**Methods:**

The study included 339 preeclamptic cases and 337 normotensive controls. Depression and depressive symptoms during pregnancy were assessed using the Patient Health Questionnaire (PHQ-9). Odds ratios (OR) and 95% confidence intervals (CI) were estimated from logistic regression models.

**Results:**

The prevalence of moderate depression was 11.5% among cases and 5.3% among controls. The corresponding figures for moderate-severe depression were 3.5% for cases and 2.1% for controls. Compared with non-depressed women, those with moderate depression had a 2.3-fold increased risk of preeclampsia (95% CI: 1.2–4.4), while moderate-severe depression was associated with a 3.2-fold (95% CI: 1.1–9.6) increased risk of preeclampsia. Associations of each of the 9-items of the PHQ-9 depression screening module with preeclampsia risk were also observed.

**Conclusion:**

Our findings are consistent with the only other published report on this topic. Collectively, available data support recent calls for expanded efforts to study and address depression among pregnant women.

## Background

There is mounting evidence that depression is an independent risk factor for the progression of cardiovascular disease (CVD) [1]. Many factors can contribute to an increased risk for CVD in depressed patients. The concept of a bio-behavioral model to explain the relationship between depression and CVD is gaining support in the literature [2,3]. This model includes characteristics ranging from an increased presence of classical risk factors for CVD (such as smoking, inactivity, hypertension, and diabetes) to changes in the immune system and dysregulation of the autonomic nervous system. Platelets from depressed patients have also been found to have increased 5-hydroxytryptamine 2 (5-HT2) binding density, suggesting that depressed patients may be at increased risk for serotonin-mediated platelet activation and coronary artery vasoconstriction [1].

Preeclampsia, a major cause of perinatal morbidity and mortality, occurs in 5–7% of pregnancies and is diagnosed when both hypertension and proteinuria are present [4]. Preeclampsia and CVD have overlapping risk factors, such as hypertension and diabetes, and appear to share similarities in pathophysiology, as both involve endothelial damage, vasoconstriction, platelet activation, and aggregation mediated by serotonin [5]. Because depression is an independent risk factor for the progression of CVD [1], it may also be a potential risk factor for preeclampsia. However, only one study has investigated the link between depression and preeclampsia [6]. Kurki and colleagues studied 623 pregnant nulliparous Finnish women at low risk for preeclampsia. All women had a healthy first trimester and were then evaluated using the Beck Depression and Anxiety scale at a median of 12 weeks' gestation. The authors observed that depression was associated with an increased risk of preeclampsia [odds ratio (OR) 2.5; 95% confidence interval (CI): 1.2–5.3] [6]. Taking into account the limited literature on this topic, we evaluated the relationship between maternal depression/depressive symptoms during pregnancy and preeclampsia risk, using data from a large case-control study of preeclampsia risk factors among Peruvian women.

## Methods

This case-control study was conducted at the Materno Perinatal Institute of Lima and the Dos de Mayo Hospital in Lima, Peru, from May 2004 through October 2005. Both institutions are operated by the Peruvian government and are primarily responsible for providing maternity services to low-income women residing in Lima. This study was approved by the ethical committees of both hospitals. All participants provided written informed consent.

Cases were selected from those women with a diagnosis of preeclampsia. Potential preeclampsia cases were identified by daily monitoring of all new admissions to antepartum wards, emergency room wards, and labor and delivery wards of the study hospitals. Study subjects were recruited during their hospital stay. Study personnel made periodic visits to each ward in a fixed order for the purposes of identifying potential cases and controls for the present study. Preeclampsia was defined by sustained pregnancy-induced hypertension with proteinuria [4]. Hypertension was defined as sustained blood pressures of at least 140/90 mm Hg on or after 20 completed weeks' gestation and on at least two occasions at least four hours apart. Proteinuria was defined as a urine-protein concentration ≥ 30 mg/dl (or 1+ on a urine dipstick) in at least two random specimens collected at least four hours apart. Approximately 95% of eligible cases approached and asked to participate in the study elected to do so. There were 6 eclampsia (preeclampsia further complicated by maternal seizure) cases in the current study.

Controls were women with pregnancies uncomplicated by pregnancy-induced hypertension or proteinuria. Each day during the enrollment period, controls were numbered in the order in which they were admitted and identified. Subsequently, they were approached in the order in which research personnel identified them. Of the 362 controls approached, 337 (93%) agreed to participate in the study.

A standardized, structured questionnaire was used to collect information regarding maternal sociodemographic, medical, reproductive, and lifestyle characteristics during in-person interviews. All interviews were conducted in the hospital. We used the Patient Health Questionaire-9 (PHQ-9) to assess study subjects' experience of depression or depressive symptoms during pregnancy. PHQ-9 is from the Primary Care Evaluation of Mental Disorders Patient Health Questionnaire (PRIME-MD PHQ) and it consists of 9 items of depressive symptoms plus a question about functional impairment. Each item was marked on a scale from 0 to 3, and the circled numbers were added to obtain scores ranging from 0 to 27. Maternal experience of depression was determined by the response to the question: "During the last nine months (during your pregnancy), how often have you been bothered by any of the following problems?" Responses were coded, 0 = Not at all; 1 = Several days; 2 = More than half the days; 3 = Nearly every day. The problems were as follows: (i) Little interest or pleasure in doing things; (ii) Feeling down, depressed, or hopeless; (iii) Trouble falling or staying asleep, or sleeping too much; (iv) Feeling tired or having little energy; (v) Poor appetite or eating too much; (vi) Feeling bad about yourself or feeling that you are a failure or have let yourself or your family down; (vii) Trouble concentrating on things such as reading the newspaper or watching TV; (viii) Moving or speaking so slowly that other people could have noticed. Or the opposite – being so restless that you have been moving around a lot more than usual; (ix) Thought that you would be better off dead, or hurting yourself in some way. From these nine items, we calculated the total score. We interpreted the degree of depression according to total scores as follows: Minimal = 0–4; Mild = 5–9; Moderate = 10–14; Moderate to severe = 15–19; Severe ≥ 20 [7]. In a recent validation study of the PHQ-9 questionnaire, the authors concluded that the instrument is a reliable and valid measure of depression severity and a useful clinical and research tool [7]. We also explored the association between the frequency of experiencing each depressive symptom and preeclampsia risk.

Maternal and infant records were reviewed to collect detailed information concerning antepartum, labor, and delivery characteristics, as well as conditions of the newborn. Maternal anthropometric measures (height, weight, and mid-arm circumference) were taken during participants' hospital stays. Gestational age was based on the date of the last menstrual period and was confirmed by an ultrasound examination performed before 20 weeks. Pre-pregnancy body mass index (BMI), a measure of overall maternal adiposity, was calculated as weight in kilograms divided by height in meters squared. Women were classified as lean (BMI < 19.8 kg/m^2^), normal (BMI = 19.8–26.0 kg/m^2^), overweight (BMI = 26.1–29.0 kg/m^2^) and obese (BMI > 29.0 kg/m^2^), based on the Institute of Medicine's criteria [8].

We examined the frequency distribution of maternal sociodemographic characteristics and reproductive histories according to case and control status. Initial univariate analyses were carried out in order to determine unadjusted odds ratios (ORs) and 95% confidence intervals (CIs). Effect modification was evaluated by stratified analyses and by including appropriate interaction terms in logistic regression models [9]. If there appeared to be no effect modification, logistic regression procedures were used to simultaneously control for confounding variables while estimating ORs and 95% CIs. Confounders were defined as those factors that altered unadjusted ORs by at least 10%. Final logistic regression models included confounders, as well as those covariates of *a priori *interest (i.e., maternal age and parity). All analyses were performed using STATA 9.0 statistical software (Stata, College Station, Texas, USA). All continuous variables are presented as mean ± standard deviation (SD). All reported p-values are two-tailed, and all confidence intervals were calculated at the 95% level.

## Results

Sociodemographic and reproductive characteristics of cases and controls are presented in Table 1. Compared with controls, cases tended to be older, single, heavier, and nulliparous. They were also more likely to be employed during pregnancy. Preeclampsia cases were more likely to deliver preterm term and to deliver by cesarean section.

**Table 1:** Sociodemographic and Reproductive Characteristics of the Study Population, Lima, Peru, 2004–2005

Characteristics	Preeclampsia Cases (N = 339)		Control Subjects (N = 337)		P-value
	
	n	%	n	%	
Maternal age at delivery (years)	27.0 ± 7.1*		25.7 ± 5.8		0.01
Maternal age at delivery (years)					
< 20	44	13.0	34	10.1	0.003
20–34	229	67.5	265	78.6	
≥ 35	66	19.5	38	11.3	
Maternal education ≤ 12 years	289	85.3	300	89.0	NS
Single married status	81	24.2	45	13.4	< 0.01
Employed during pregnancy	134	39.5	98	29.1	0.02
Nullipara	160	47.2	117	34.7	< 0.05
No prenatal vitamins	68	20.1	59	17.5	NS
Gestational age at delivery (weeks)	37.4 ± 3.1 *		39.0 ± 2.2		< 0.001
< 28	6	1.8	3	0.9	< 0.001
28–33	35	10.3	7	2.1	
34–36	48	14.2	11	3.2	
≥ 37	250	73.7	316	93.8	
Infant birth weight (grams)	2852.9 ± 830.6 *		3248.2 ± 621.1		< 0.001
Pre-pregnancy body mass index (kg/m^2^)	24.8 ± 4.4 *		23.1 ± 3.3		< 0.001
Pre-pregnancy body mass index (kg/m^2^)					
< 19.8 (lean)	32	9.4	29	8.6	< 0.001
19.8–26.0 (normal)	182	53.7	245	72.7	
26.1–29.0 (overweight)	60	17.7	34	10.1	
> 29.0 (obese)	53	15.6	16	4.7	
Missing	12	3.5	13	3.9	
Delivery mode					
Vaginal delivery	108	31.9	318	94.4	< 0.001
Cesarean delivery	230	67.9	19	5.6	

We employed a two-sample Student's t-test to compare the continuous variables between preeclampsia cases and controls; and Chi-square tests or Fisher's exact tests were used to compare the categorical variables between the two groups.

NS = not statistically significant.

* Mean ± SD (standard deviation)

The prevalence of moderate depression (score 10–14) was 11.5% among preeclamptic women and 5.3% among normotensive controls (Table 2). The corresponding figures for moderate to severe depression (score 15–19) were 3.5% for cases and 2.1% for controls. Compared with minimally depressed women (score 0–4), those with moderate depression during pregnancy had a 2.6-fold increased risk of preeclampsia (OR = 2.6; 95% CI: 1.4–4.6). The association was attenuated slightly after adjusting for maternal age, parity, and pre-pregnancy body mass index (OR = 2.3; 95% CI: 1.2–4.4). Moderate to severe depression was associated with a 3.2-fold (95% CI: 1.1–9.6) increased risk of preeclampsia after adjusting for confounders. An extremely small number of subjects (two preeclampsia cases and no normotensive controls) with severe depression prohibited further study of this spectrum of depression with preeclampsia risk.

**Table 2:** Odds Ratios (OR) and 95% Confidence Intervals (CI) for Preeclampsia According to Maternal History of Depression during Pregnancy, Lima, Peru, 2004–2005

Maternal History of Depression (total score)	Preeclampsia Cases (N = 339)	Control Subjects (N = 337)	Unadjusted OR (95% CI)	Adjusted OR (95% CI)
	
	n (%)	n (%)		
Minimal (< 4)	180 (53.1)	213 (63.2)	1.0 (referent)	1.0 (referent)
Mild (5–9)	106 (31.3)	98 (29.1)	1.3 (0.9–1.8)	1.2 (0.9–1.8)
Moderate (10–14)	39 (11.5)	18 (5.3)	2.6 (1.4–4.6)	2.3 (1.2–4.4)
Moderate-severe (15–19)	12 (3.5)	7 (2.1)	2.0 (0.8–5.3)	3.2 (1.1–9.6)
Severe (≥ 20)	2 (0.6)	0 (0.0)	---	---
Missing	0 (0.0)	1 (0.3)		
	p-value for trend		0.001	0.001

Adjusted by maternal age (continuous), nulliparity (yes vs. no), maternal overweight status (pre-pregnancy body mass index ≤ 26.0 vs. > 26.0 kg/m^2^).

Table 3 shows associations between the frequency of each of the depressive symptoms and preeclampsia risk. Each depressive symptom was associated with increased preeclampsia risk. However, we did not observe trends in risk across increasing frequency of any of the seven symptoms. For example, women who experienced little interest or pleasure in performing activities during several days of their pregnancies was at 2.5-fold increased risk compared with women who did not report this symptom (OR = 2.5; 95% CI: 1.7–3.7). Women who experienced this symptom for more than half the pregnancy were at roughly similarly increased risk (OR = 2.2; 95% CI: 1.1–4.4).

**Table 3:** Odds Ratios (OR) and 95% Confidence Intervals (CI) for Preeclampsia According to Maternal History of Depressive Symptoms during Pregnancy as Measured by the PHQ-9 Item Questionnaire, Lima, Peru, 2004–2005

PHQ-9 Items	Preeclampsia Cases (N = 339)	Control Subjects (N = 337)	Unadjusted OR (95% CI)	Adjusted OR (95% CI)
Little interest or pleasure in doing things				
Not at all	185	247	1.0 (referent)	1.0 (referent)
Several days	128	68	2.5 (1.8–3.6)	2.5 (1.7–3.7)
More than half the days	26	21	1.7 (0.9–3.0)	2.2 (1.1–4.4)
Nearly every day	0	1	---	---
Feeling down, depressed, or hopeless				
Not at all	92	141	1.0 (referent)	1.0 (referent)
Several days	101	40	3.9 (2.5–6.1)	3.7 (2.3–5.9)
More than half the days	142	153	1.4 (1.0–2.0)	1.3 (0.9–1.9)
Nearly every day	4	3	2.0 (0.5–9.3)	---
Trouble falling or staying asleep, or sleeping too much				
Not at all	184	220	1.0 (referent)	1.0 (referent)
Several days	73	36	2.4 (1.6–3.8)	2.4 (1.5–3.8)
More than half the days	77	79	1.2 (0.8–1.7)	1.2 (0.8–1.8)
Nearly every day	5	2	3.0 (0.6–15.6)	3.3 (0.6–18.8)
Feeling tired or having little energy				
Not at all	168	206	1.0 (referent)	1.0 (referent)
Several days	93	58	2.0 (1.3–2.9)	1.8 (1.2–2.7)
More than half the days	76	71	1.3 (0.9–1.9)	1.3 (0.9–2.0)
Nearly every day	2	2	1.2 (0.2–8.8)	1.2 (0.2–10.1)
Poor appetite or eating too much				
Not at all	176	227	1.0 (referent)	1.0 (referent)
Several days	75	51	1.9 (1.3–2.9)	1.8 (1.2–2.8)
More than half the days	80	57	1.8 (1.2–2.7)	1.7 (1.1–2.6)
Nearly every day	8	2	5.2 (1.1–24.6)	4.6 (0.9–23.4)
Feeling bad about yourself – or that you are a failure or have let yourself or your family down				
Not at all	262	300	1.0 (referent)	1.0 (referent)
Several days	58	27	2.5 (1.5–4.0)	2.3 (1.4–3.9)
More than half the days	17	10	2.0 (0.9–4.3	2.1 (0.9–5.2)
Nearly every day	2	0	---	---
Trouble concentrating on activities such as reading the newspaper or watching TV				
Not at all	274	302	1.0 (referent)	1.0 (referent)
Several days	37	18	2.3 (1.3–4.1)	2.4 (1.3–4.6)
More than half the days	26	16	1.8 (0.9–3.4)	1.8 (0.9–3.7)
Nearly every day	2	1	2.2 (0.2–24.4)	3.0 (0.2–37.0)
Moving or speaking so slowly that other people could have noticed.				
Not at all	307	323	1.0 (referent)	1.0 (referent)
Several days	15	7	2.3 (0.9–5.6)	2.0 (0.8–5.4)
More than half the days	15	6	2.6 (1.0–6.9)	3.2 (1.1–9.1)
Nearly every day	2	0	---	---
Missing	0	1		
Thought that you would be better off dead or hurting yourself in some way				
Not at all	260	276	1.0 (referent)	1.0 (referent)
Several days	36	37	1.0 (0.6–1.7)	0.9 (0.5–1.5)
More than half the days	41	24	1.8 (1.1–3.1)	1.8 (0.99–3.3)
Nearly every day	2	0	---	---
Degree of difficulty of daily living if you had any of above symptoms				
Not difficult at all	175	183	1.0 (referent)	1.00 (referent)
Somewhat difficult	108	49	2.3 (1.6–3.4)	2.2 (1.4–3.3)
Very difficult	18	10	1.9 (0.9–4.2)	2.3 (0.9–5.7)
Extremely difficult	0	0	---	---
Unknown or N/A	38	95		

Adjusted by maternal age (continuous), nulliparity (yes vs. no) and maternal pre-pregnancy overweight status

## Discussion

Our findings of associations between depression and preeclampsia in Peruvian women are consistent with the only other published report on this topic, based on a study of Finnish women [6]. We report that the individual depression symptoms appear to be generally similar in the strength of their relationship to preeclampsia. We were unable to observe a trend of increased preeclampsia risk with frequency of depressive symptoms because we were limited by the low number of responses in the category of the highest frequency (nearly everyday).

Traditionally, pregnancy is considered a period of well-being and happiness, and was once thought to protect women from depression [10]. However, women of child-bearing age frequently suffer from major depression [11,12]. The prevalence of moderate to severe depression in our self-report screen module was 15.6% in preeclampsia cases and 7.4% in control subjects. These frequencies are generally consistent with the meta-analysis report by Gaynes et al [12], in which the authors reported that the combined estimates of point prevalence ranged from 8.5% to 11.0% at different times during pregnancy for major and minor depression. The authors summarized data from a large number of studies that used a wide variety of depression screening and diagnostic instruments including the Beck Depression Inventory (BDI) and the Center for Epidemiologic Studies Depression Scale (CES-D). Findings across studies were reasonably similar despite different depression assessment approaches and characteristics of study populations.

Our finding of an increased risk of preeclampsia with maternal depression is consistent with numerous reports that focused on the occurrence of cardiovascular diseases in depressed non-pregnant women and men [13,14]. Clinically diagnosed major depression has been shown to be associated with more than doubling in risk of CVD (pooled OR: 2.54, 95% CI: 2.07–3.10) [13]. Additionally, investigators have reported that depressed mood is associated with increased risks of myocardial infarction, and coronary heart disease (odds ratios ranging from 1.43 to 1.63) [13,14].

Several potential limitations should be taken into consideration when interpreting the results of this study. First, our analyses are based on cross-sectionally collected data, which may be subject to recall bias. There has been one longitudinal study of Finnish women [6]; however, more longitudinal studies are needed to re-examine the potential causal relation between maternal experience of depression and preeclampsia risk in different populations. Second, we used a depression screening instrument to categorize participants according to depression/depressive symptoms. Participants did not have formal diagnostic examinations. As a result, some misclassification is possible. However, Wulsin et al. [15] reported that the PHQ-9 proved easy to administer to a population of poor, young Honduran women who had never participated in research before and were similar to Peruvian women included in our study. The PHQ-9 results also showed a sensitivity of 77% and a specificity of 100% compared with the Structured Clinical Interview for DSW-IV mood disorder module. In addition, our assessment of maternal depression and depressive symptoms was limited to the duration of the pregnancy. We did not collect information to allow for the accurate sub-classification of mild and severe preeclampsia. This limitation also hindered our capability to further assess the correlation between severity of depression and severity of preeclampsia. Last, although we adjusted for multiple confounding factors, as with all observational studies, we cannot exclude the possibility of some residual confounding.

Associations between depression and increased risk of preeclampsia are biologically plausible. Depression has been associated with hypothalamic-pituitary-adrenal (HPA) axis hyperactivity [16]. Maternal depression is thought to activate the mother's HPA axis and, in turn, is regulated by peptides derived from the activated HPA axis. Depression may cause an increase in the release of corticotropin-releasing hormone (CRH) from the placenta via the actions of catecholamines and cortisol [17-20]. Smith and colleagues examined the relationships between mood changes, obstetric experience, and alterations in plasma cortisol, beta-endorphin, and CRH in 97 women [20]. Plasma levels of these hormones were obtained throughout pregnancy, at delivery, and postpartum. Smith and colleagues reported that the prevalence of mood disturbances found in the late antenatal period was higher than the level found in the postnatal period; the prevalence correlated with hormone levels, which peaked in late pregnancy and fell postpartum [20]. These data suggest a role for CRH and the HPA axis in the relationship between antenatal mood states and obstetric events. Depression may be mediated by an altered excretion of vasoactive hormones and other neuroendocrine transmitters. This may in turn cause vasoconstriction and uterine artery resistance and, therefore, elevate blood pressure [6,19]. Recent evidence also indicates the involvement of pro-inflammatory cytokines in the pathogenesis of major depression. The stress hormones may facilitate inflammation through induction of interleukins (IL) such as IL-1, IL-6, IL-8, IL-18, tumor necrosis factor-alpha, and C-reactive protein production [21].

## Conclusion

Our results, combined with those reported by others [6], suggest that the risk of preeclampsia is increased in women experiencing depression during pregnancy. Available research suggests that depression is one of the most common complications of pregnancy, and that fairly accurate and feasible screening measures are available [22-24]. Longitudinal cohort studies, with prospective clinical assessment of depression, and studies in ethnically and racially diverse populations are warranted.

## Competing interests

The author(s) declare that they have no competing interests.

## Authors' contributions

SES, NL, PG and MA conceived the study and participated in study design. NL and PG contributed to the acquisition of clinical data. MW and CQ completed statistical analyses. All authors participated in interpreting study findings and drafting the manuscript. All authors read and approved the final manuscript.

## Pre-publication history

The pre-publication history for this paper can be accessed here:


